# Scaling up breastfeeding policy and programs in Samoa: application of the Becoming Breastfeeding Friendly initiative

**DOI:** 10.1186/s13006-019-0245-6

**Published:** 2020-01-06

**Authors:** Christina Soti-Ulberg, Amber Hromi-Fiedler, Nicola L. Hawley, Take Naseri, Analosa Manuele-Magele, John Ah-Ching, Rafael Pérez-Escamilla, on behalf of BBF Samoa Committee, Maria Sanele, Namulauulu Tautala Maualaivao, Visesio Faaleaga, Saunimaa Aiolupotea, Jyothi Alex Abraham, Sina Tuautu, Cedrela Tamati, Patricia Lesatele, Naomi Harris, Samasoni Moala, Siaosi U. Leleimalefaga, Lusi Faleupolu, Ulai Tapa Fidow, Acquin Time Fiu, Avai’a Tuilaepa Lautusi, Robyn Roache, Momoti Ulisese Tapuvae, Vaelei Apelu, Selaupasene Ualesi, Christian Atoa

**Affiliations:** 1 Ministry of Health, Apia, Samoa; 2 Yale School of Public Health, 60 College Street, New Haven, CT USA

**Keywords:** Breastfeeding, Samoa, Becoming Breastfeeding Friendly, Scaling up

## Abstract

**Background:**

Breastfeeding is a critical, evidence-based intervention that addresses malnutrition, improves early childhood development outcomes, and influences long-term maternal and infant health by reducing the non-communicable disease risk. Scaling up breastfeeding is an indisputably strong action countries can take to prevent suboptimal maternal and infant health outcomes. The Becoming Breastfeeding Friendly (BBF) initiative assists countries with scaling up breastfeeding policy and programs. BBF has been successfully implemented within Latin America, Africa, Europe and South-East Asian regions. This study assessed its application in Samoa.

**Methods:**

In 2018, BBF was implemented in Samoa by a 20 member committee of breastfeeding experts who participated in collecting and utilizing national level data to score the degree of friendliness of Samoa’s breastfeeding environment, identify gaps, and propose policy recommendations to address those gaps. This eight-month process resulted in a public event where priority recommendations were widely disseminated to decision makers and actions agreed upon.

**Results:**

The total BBF Index score for Samoa was 1.6 out of 3.0, indicating a moderate breastfeeding friendly environment for scaling up policies and programs that protect, promote, and support breastfeeding. Gear total scores indicated that seven of the eight gears were moderately strong within Samoa, while the eighth gear, funding and resources, was weakest in strength. Six prioritized recommendations emerged: 1) development and implementation of a National Breastfeeding Policy and Strategic Action Plan; 2) strengthening monitoring and evaluation of all breastfeeding activities; 3) ratifying the International Labour Organization’s Maternity Protection Convention 2000 (No 183); 4) identifying high-level advocates to champion and serve as role models for breastfeeding; 5) creation of a national budget line for breastfeeding activities; and 6) hiring of a national breastfeeding coordinator and trainer. Decision makers demonstrated commitment by signing the breastfeeding policy for hospitals ahead of the BBF dissemination meeting and electing to move forward with establishing lactation rooms within government ministries.

**Conclusion:**

Implementation of BBF in Samoa yielded important policy recommendations that will address current gaps in national level breastfeeding support. The BBF consultation process can be successfully applied to other countries within the Western Pacific region in order to strengthen their breastfeeding programs.

## Background

Breastfeeding is a ‘triply-duty action’ that has demonstrated immense potential as an evidence-based intervention to address malnutrition in all its forms (i.e. underweight, stunting, micronutrient deficiencies, overweight, and obesity) while also improving early childhood development outcomes [1]. Undeniably, the early benefits of breastfeeding extend throughout the life course to protect the health of a mother and her infant further by reducing the longer term risk of certain cancers and other chronic diseases [2]. For countries that face considerable concerns associated with the presence of multiple forms of malnutrition plus increasing prevalence of non-communicable diseases (NCDs), scaling up national breastfeeding policies and programs can be a cost-saving strategy [3] to avoid overwhelming health care costs associated with these conditions [4]. Indeed, the cost of inaction (i.e. not breastfeeding) can lead to significant human capital, economic, and environmental loses for a country [5]. Unfortunately, countries committed to scaling up breastfeeding friendly environments have been challenged by frameworks that are limited and lack effectiveness as well as few valid decision-making tools [6].

Becoming Breastfeeding Friendly (BBF) is an evidence-informed initiative that addresses this gap by providing countries with a valid toolbox to assess and develop a plan to scale up national policies and programs to strengthen the breastfeeding friendly enabling environment [7]. Becoming Breastfeeding Friendly is grounded in the Breastfeeding Gear Model framework, which uses a complex adaptive systems approach to stipulate that eight gears - advocacy; political will; legislation & policy; funding & resources; training & program delivery; promotion; research & evaluation; coordination, goals & monitoring – must work in harmony to achieve large-scale improvements in a country’s breastfeeding friendly environment [8]. The BBF toolbox consists of a BBF index (BBFI), case studies, and a 5-meeting process [9]. Application of the BBF toolbox is expected to drive policy changes within a country, subsequently strengthening the breastfeeding environment following the cyclical policy heuristic model that includes five key elements: (a) leadership and partnerships; (b) agenda setting; (c) policy formulation and adoption; (d) implementation; and (e) evaluation [9, 10]. Ideally, by strengthening each of these elements, the BBF toolbox contributes to strengthening the friendliness of the breastfeeding environment through its repeated application across time [9, 10].

Over the past few years, BBF has been successfully implemented in countries in Latin America [11], Africa [12], and Europe [13, 14]. Yet, until last year, it had not been applied to a country in the Western Pacific region. Samoa served as an ideal setting to pilot the implementation of BBF within that region because of the country’s ongoing partnership with the BBF lead university, Yale, and their commitment to maternal child health research and policy making. Indeed, the country had already demonstrated commitment to improving breastfeeding outcomes, as evidenced by the prominence of breastfeeding promotion in their National Health Strategy [15]. Plus, they had expressed a need to receive guidance on how to strategically move forward improving the coverage and quality of their breastfeeding programs.

Application of BBF in Samoa has wide-reaching implications for the Western Pacific region. Although breastfeeding is highlighted in many country health strategies and in the broader Action Plan for Healthy Newborn Infants in the Western Pacific Region [16], the prevalence of exclusive breastfeeding among infants less than 6 months of age varies widely across the Western Pacific region from more than 70% in the Solomon Islands to less than 40% in Tuvalu [17]. Guided by the World Health Organization regional office and the Secretariat of the Pacific Community, the Pacific Islands have demonstrated success in developing Pacific-wide strategies and monitoring plans for NCDs and infectious diseases. Following the implementation of BBF in Samoa, there is the potential for Samoa to take a leadership role in the dissemination of BBF to other Pacific nations and the development of a Pacific-wide strategy on breastfeeding promotion. Thus, the overarching objective of this manuscript is to describe the piloting and feasibility assessment of the BBF toolbox in Samoa, and document lessons learned for potential further dissemination in this region.

## Methods

### Study site

Samoa is an upper-middle income country located in the Western Pacific region with a population of ~ 197,000 and 6100 births each year [18]. Polynesia as a whole, and Samoa specifically, are recognized for their extremely high burden of chronic disease (93% of women aged 25–64 are overweight or obese) [19] and concurrent issues of maternal and child malnutrition (one-third of women of reproductive age are anemic, 29% of children aged 2–5 years are stunted, and 34% anemic) [20], both of which may be positively impacted by improving breastfeeding practices. While uptake of breastfeeding is high (94% of all infants are breastfed) and median duration of breastfeeding is 21 months, exclusive breastfeeding declines after 2 months of age from 75 to 55% at 4–5 months [18].

### BBF implementation in Samoa

The Samoan Ministry of Health (MOH) led the implementation of BBF between January–August 2018 on behalf of the Samoan government. Beginning in January 2018, preparations began with sensitizing key stakeholders to BBF, identifying a director to lead BBF implementation in Samoa, and identifying country committee members to participate in the 5-meeting process. In consultation with other government agencies (the Ministry of Women, Community, and Social Development (MWCSD), Ministry of Education, Sports, and Culture (MESC), Ministry of Commerce, Industry, and Labor (MCIL), and the Public Service Commission (PSC)) invitation letters to participate in the Samoan BBF country committee were sent by the MOH to the CEOs and heads of eight organizations representing government, academia, and non-governmental organizations. A country committee of 20 breastfeeding, lactation, and/or women’s health experts from eight institutions (MOH, MCIL, PSC, MESC, Samoa Family Health Association, Samoa Red Cross, National University of Samoa, National Health Services) was established before the 5-meeting process began.

In Samoa, the 1st meeting represented the formal in-depth introduction of BBF to the BBF country committee members as well as government ministries and nongovernment key stakeholders. The 1st meeting educated BBF committee members about the BBFI, which is the primary assessment tool that measures the strength of a country’s breastfeeding enabling environment through 54 individual benchmarks [7]. The 1st meeting also introduced participants to subsequent toolbox components, with the goal of empowering them to successfully implement BBF in their country [7]. Over 23 individuals attended, including the BBF Samoa co-investigator from Yale University (NLH), who also participated in most subsequent BBF meetings. Three aims were achieved during this 2-day meeting: a) introduced the current breastfeeding environment in Samoa, b) described the BBF toolbox components in detail, and c) initiated the assessment of the degree of friendliness of the breastfeeding environment with the BBFI. The latter included the formation of four teams of BBF committee members (5 members each) who worked on developing plans to gather data for the specific gears they were assigned to assess.

Following the first meeting, the four teams (i.e. gear teams) were tasked with identifying, collecting, and documenting local, district and national level data (i.e. national strategies, policies, legislation, survey data, media articles) relevant to scoring the benchmarks within their allocated gears. Gear teams met to discuss the data plus assign preliminary scores to their benchmarks. Indeed, each gear team held between two to five meetings throughout the 5-meeting process to ensure benchmarks were fully assessed, scores assigned, and recommendations developed.

The BBF country committee reconvened 2 months later. At this 2nd meeting all gear teams, except the training & program delivery gear team, presented their preliminary benchmark scores to the full committee. By the second meeting, the training & program delivery gear team had not fully scored the requisite benchmarks and subsequently did not have scores to present. Instead, they presented their initial benchmark and gear scores at the 3rd meeting.

Across the seven gear team presentations at the 2nd meeting, the discussion centered around finding out: a) whether the benchmark scores reflected the available data, b) adequacy and gaps in the collected data, c) if consensus could be reached on the benchmark scores given the existing data, d) if additional data were required to reach consensus on the benchmark scores, and e) if any modifications in the initial recommendations were required at this point. In situations where the committee felt more data was required to score a benchmark, additional data was sought through in-depth interviews, reports, and other available sources. The quality of the data was considered by the committee during discussion and when reaching consensus on benchmark scores.

Following the 2nd meeting, gear teams independently obtained the remaining data, confirmed as well as adjusted benchmark scores based on available, additional collected data, and developed recommendations based on identified benchmark gaps. Benchmark scores for all gears were presented at the 3rd meeting in June 2018 where the BBF committee reached final consensus on the benchmark scores and subsequently calculated the gear scores as well as the total BBF score. Detailed recommendations were presented, discussed by gear, and revised to reflect the discussion.

In preparation for the 4th meeting, the BBF Samoa director (CSU) followed a multistep process developed by the BBF team at Yale to assist BBF committees with prioritizing their recommendations based on three key grading criteria: 1) effectiveness (i.e., will the recommendation have an effect or impact on breastfeeding outcomes), 2) affordability (i.e. is the recommendation affordable and are there the financial means to pay for it taking into account available information on the cost of implementing such a recommendation), and 3) feasibility (i.e. are all the necessary resources to implement such a recommendation present). Initially, the director combined recommendations that were duplicated across gears and then used the BBF case studies from the toolbox to provide supporting evidence for the recommendations. An online google survey was created and distributed to BBF committee members to answer questions to grade the effectiveness, affordability, and feasibility of each recommendation. Responses were collected, summed, and averaged so that each recommendation had an average score for each criterion (i.e. effectiveness, affordability, and feasibility) plus an overall average score.

The BBF committee reconvened 3 weeks later for the 4th meeting. At this one-day meeting, the director presented the survey results for each recommendation to the BBF committee. After an in-depth discussion, the committee felt the recommendations could be grouped into themes. Therefore, consensus was reached on the top six themes to represent their top overarching priority recommendations. Action plans for each prioritized recommendation were also developed to guide the future implementation of the recommendations. Discussion also ensued to identify which actions could be immediately addressed.

After the meeting, the prioritized recommendations and action plans were circulated to BBF committee members to finalize them. A one-page infographic was developed by the MOH nutrition unit under the director’s supervision and contained a brief description of the: a) breastfeeding environment in Samoa prior to BBF implementation, b) BBF initiative, c) gear scores, and d) six prioritized recommendations. Additionally, a four-page policy brief was also written by the Samoa leadership team in collaboration with the BBF team from Yale University to describe in more detail each topic within the infographic. Proposed actions were also included for each prioritized recommendation. Invitation letters were sent to government and non-government leaders as well as media personnel to attend the 5th meeting when BBF findings and policy recommendations would be disseminated.

On August 1, 2018, findings from the BBF initiative were disseminated in Samoa through a day long public event. The timing coincided perfectly with the beginning of World Breastfeeding Week and elicited attendance from over 100 individuals including high level decision makers from government ministries and health services, non-governmental organizations, and academia. The BBF PI and BBF Samoa co-investigator based at Yale University (RPE and NLH, respectively) also attended. The high level of government support was evident with the key note address being delivered by the Associate Minister of Health. The infographic and policy briefs were disseminated during the event to support the presentation of the findings and generate feedback and discussion among policymakers to reach consensus on the way forward.

## Results

### BBFI gear scores

**Fig. 1:**
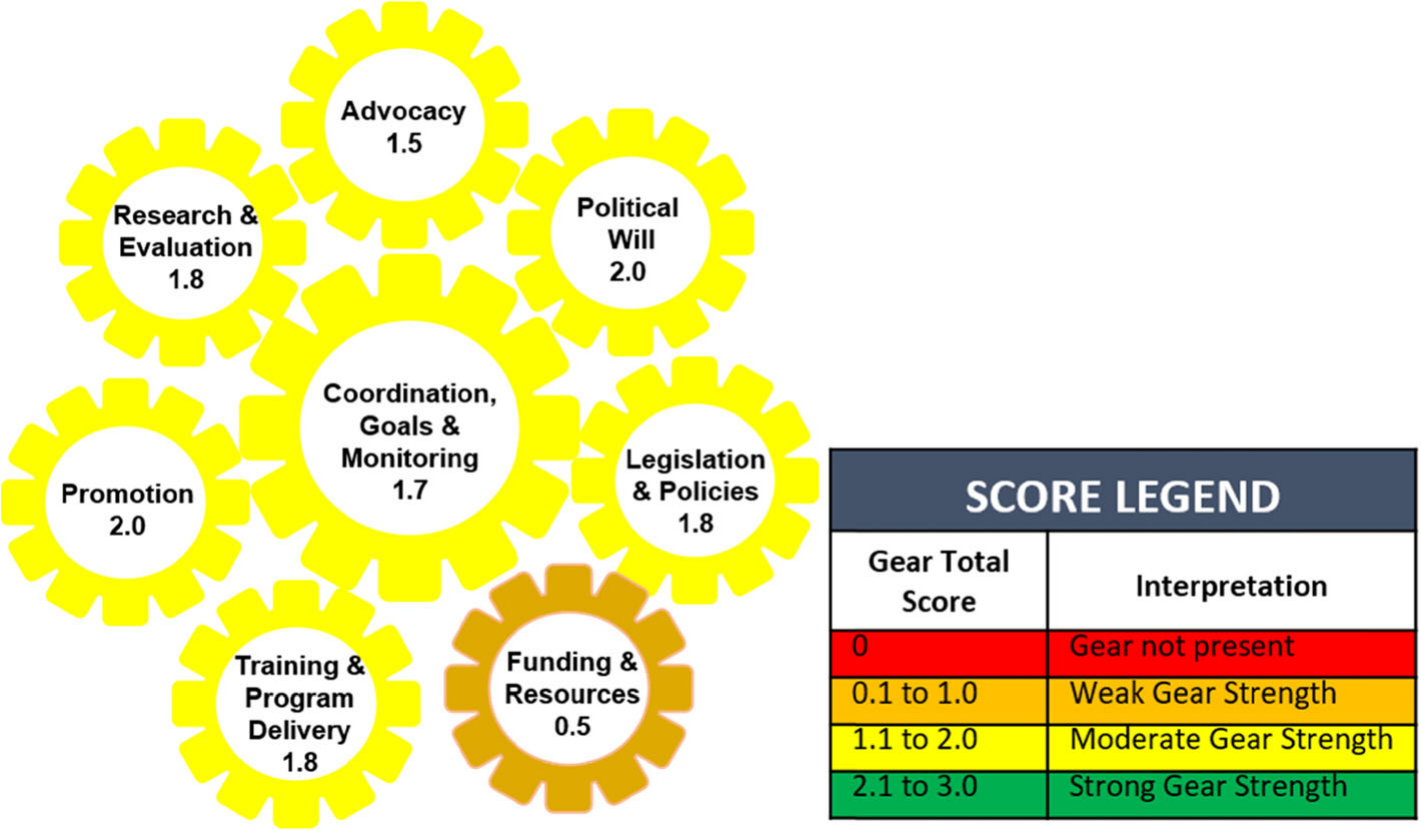
The final eight gear scores for the Becoming Breastfeeding Friendly initiative in Samoa

The total BBFI score for Samoa was 1.6 out of a maximum of 3.0, indicating that the country had a moderately friendly breastfeeding environment for scaling up policies and programs that protect, promote, and support breastfeeding. Supporting this finding, the gear total scores indicated that seven of the eight gears were moderately strong within Samoa (Fig. 1). The funding and resources gear was the only gear that was assessed as being weak, which was primarily due to: a) lack of a national budget line for breastfeeding, b) lack of adequate funding and human resources for breastfeeding, c) lack of a national breastfeeding coordinator that is funded full time to primarily work on breastfeeding promotion, and d) having a limited formal mechanism for funding of maternity entitlements (Table 1).

**Table 1:** BBF gear score, gear strength, gaps and recommendations. Samoa, 2018

Gear	Gear Score	Gear Strength	Gaps	Recommendations
Advocacy	1.5	Moderate	• Lack of any high-level advocates or influential individuals who have taken on breastfeeding as a cause that they are promoting • Lack of a formal National Breastfeeding Advocacy Strategy policy but some components are included in the National Nutrition Strategy (that was developed based on existing data and has indicators of effectiveness) • There is a multisectoral breastfeeding committee that has national coverage but the strengths of the political commitment are not clear or documented	• Develop an evidence-based National Breastfeeding Advocacy Strategy • Integrate breastfeeding into other health events during the year • Seek a champion on breastfeeding to become a role model to advocate for breastfeeding
Political Will	2.0	Moderate	• Expressed political commitment is only showcased during annual events; Samoan culture/traditional beliefs and values as well as the political environment (e.g. Samoan political sphere is male dominate and older) may explain why there is little, consistent public official support • Limited breastfeeding awareness at the political level	• Strengthen commitment to breastfeeding action at the political level • Improve government’s efforts towards promoting an enabling environment for breastfeeding at a national level • Government to be more proactive in creating awareness of legislations and policies affecting female employees
Legislation & Policy	1.8	Moderate	• Lack of a national breastfeeding policy, but breastfeeding is integrated into a National Health services policy • No approval yet to pass the law to enforce penalties for breaching the Code • Maternity protection laws are largely compliant with the Maternity Protection Convention 2000, but it has not been ratified • Lack of legislation for workplace accommodations for breastfeeding	• Endorse and implement legislation on infant and young child feeding and the Code • Ratify Maternity Protection Convention 2000 • Have legislation that promotes as upportive worksite for breastfeeding women • Develop and endorse a National Breastfeeding Policy • Have a formal mechanism for maternity entitlement for all working women
Funding & Resources	0.5	Weak	• Lack of a national budget line for breastfeeding • Lack of adequate funding and human resources for breastfeeding; • Lack of a national breastfeeding coordinator that is funded full time to primarily work on breastfeeding promotion • Limited formal mechanism by which maternity entitlements are funded	• Create a national budget line for breastfeeding promotion activities. • Have a national coordinator position for breastfeeding for lactation counselors/ master trainers and BFHI/Ten Steps
Training & Program Delivery	1.8	Moderate	• Lack of standard breastfeeding training in preservice medical, nursing, and midwife curricula • Limited number of trainers - there are two “master trainers” with only 60% of staff trained in the National Health Service with the 20 h World Health Organization breastfeeding training program • Need online breastfeeding courses/topics that are endorsed • Need for continue education credit courses for nurses, doctors, and midwives • BFHI audit reports are internal and not all reports are published online (such as on the MOH website) • Lack of any lactation consult/specialist supervisors	• Certification of the breastfeeding counseling training program by the Samoa Qualification Attorney (SQA) and MOH • Pre-service curriculum should include breastfeeding and be uniform for all preservice provider education including nursing and medicine • Have an annual breastfeeding training and regular in-service training for new staff in the maternity services
Promotion	2.0	Moderate	• Lack of a national breastfeeding promotion strategy, but rely on the National Nutrition policy • Limitations with consistent data collection and the coordination/awareness of various programs to support breastfeeding. Every organization is doing something for, breastfeeding but there is lack of coordination	• Review outdated National Infant and Young Child Feeding (IYCF) Strategy and incorporate apromotion strategy for breastfeeding • National IYCF has a set annual plan for activities to be in place
Research & Evaluation	1.8	Moderate	• MOH data is collected yearly (including annual audited BFHI reports) but it is not publicly available • Lactation counseling happens at individual basis but no monitoring system is in place	• Publish data on breastfeeding practices online via Ministry of Health website • Establish an annual population-based surveillance or monitoring system to track breastfeeding /lactation counselling and support • Strengthen implementation of routine data collection on breastfeeding during clinical visits
Coordination, Goals & Monitoring	1.7	Moderate	• Samoa has a National Multisectoral Committee on Breastfeeding with team members from MCIL, MNRE, MOH, NHS, PSC, MPE, SFH, and SRC but there is no workplan • There is insufficient information online and data are disaggregated. No e-health system is available to evaluate the breastfeeding program progress	• Conduct an Annual National Symposium for Breastfeeding • Create support network and service for breastfeeding free short message service (SMS) helpline.

### Prioritized recommendations

Fifteen initial recommendations were generated at the second meeting based on the gaps identified by BBF committee members. Following the third meeting, those recommendations were expanded into twenty- three specific recommendations (Table 1). At the fourth meeting, six themes emerged from the 23 recommendations, representing the top priority recommendations.

The committee prioritized first the development and implementation of a National Breastfeeding Policy and Strategic Action Plan. The committee recommended that a National Breastfeeding Policy be drafted to include a strategic action plan and performance indicators. The committee felt several of the recommendations were important action items within this overarching recommendation. Specifically, the strategic plan needed to include actions on the: a) integration of breastfeeding into other health events during the year, b) acknowledgement of the endorsed Infant and Young Child Feeding Regulations and an annual work plan for related activities, c) development of an annual national symposium for breastfeeding, d) creation of a support network and service for breastfeeding free text messaging hotline, e) acknowledgement of breastfeeding champion(s) to highlight their important actions and facilitate support, and f) inclusion of all people in Samoa, including potentially vulnerable groups (e.g. disabled, abused, refugees, and people living with HIV). To ensure the policy and strategic action plan received support, committee members proposed that consultations be conducted with the MOH policy division on the feasibility of drafting a policy.

The committee prioritized strengthening the monitoring and evaluation of all breastfeeding activities. The proposed actions to build this capacity included: a) making data publicly available, specifically publishing collected data on breastfeeding practices online via websites such as the Ministry of Health, b) establishing a population-based surveillance or monitoring system to track breastfeeding/lactation counselling and support every 5 years, and c) strengthening routine data collection of breastfeeding outcomes during clinical and community outreach visits and provide quarterly and annual reporting.

For Samoa, ratifying the International Labour Organization’s Maternity Protection Convention 2000 (No 183) [21] was also a top priority. The committee urged policymakers to discuss and approve provisions in the Maternity Protection Convention, especially: a) maternity protection against work that presents risks to the mother or child’s health, b) maternity leave that is more than 14 weeks with 6 weeks compulsory leave after childbirth, c) prenatal period of leave that is extended between the presumed date of birth and actual date without reducing the compulsory portion of postpartum leave, d) extending cash benefits and employment protection, and 5) establishing breastfeeding breaks at work. To strengthen the breastfeeding friendly environment in Samoa, the committee also felt that the government should establish a formal mechanism that would provide maternity entitlements for all working women. Essentially, it was suggested that the government subsidize maternity leave for the private sector to extend the current maternity leave benefits provided to these mothers from 4 weeks to 8 weeks.

The committee assessment identified several gaps related to the advocacy gear with the primary gap being that there were no high level, non-governmental advocates or influential individuals who had taken on breastfeeding as a cause that they are promoting. Thus, one of the committee’s top recommendations was to identify high level advocates that will champion and serve as role models for breastfeeding. Specifically, the committee felt it was important to take steps to: a) identify local and global champions, b) train and involve champions in breastfeeding-related activities, and c) share success stories with the community via different media outlets (e.g., TV, documentary, Radio and/or Social Media/Facebook).

The committee prioritized two recommendations to strengthen the major gaps found in the funding & resources gear. First, the committee determined that the creation of a national budget line for breastfeeding activities was essential. The committee proposed actions to develop a proposal for a national budget line that aligns with the future National Breastfeeding Policy and Strategic Action Plan and allocates funds to specific breastfeeding activities including: a) the National Breastfeeding Program, b) the Baby Friendly Hospital Initiative (BFHI)/ Ten Steps, c) monitoring and enforcement of the International Code of Marketing of Breast Milk Substitutes, d) maternity protection, e) breastfeeding related information, education and communication campaigns and materials, and f) breastfeeding training and program delivery. Lastly, the committee proposed activities to support this priority recommendation including identifying the cost of key activities for breastfeeding advocacy, promotion, education, and training every fiscal year for allocation within the proposed budget line.

The committee also prioritized the hiring of a national breastfeeding coordinator and trainer. The committee proposed specific required activities to complete the hiring of a national breastfeeding coordinator who would be responsible for coordination of the BFHI/Ten Steps initiative as well as training of lactation counselors/master trainers. In addition to developing a job description, the committee specified the recruitment, selection, and training of a national coordinator/trainer as being essential to ensuring that this position is fully functional and that it can support the scaling up of national breastfeeding programs. The committee clearly indicated that the training-the-trainer recommended cascade had to include the following core functions: a) implement BFHI, b) in-service training for staff in maternity service, c) conduct hands-on training involving individual counseling for lactation issues, d) certify breastfeeding counseling training program by the Samoa Qualification Authority (SQA), e) coordinate professional development for breastfeeding, and f) advocate for preservice curriculum to include breastfeeding and be uniform for all relevant schools including nursing and medicine.

### Feasible action plans

At the fourth meeting, the BBF committee identified the signing of an updated and revised breastfeeding policy for hospitals (originally a policy in 1995) as an immediate action that could begin to address some of the gaps.

### Commitments from decision makers

Consensus was reached during the public event that the six prioritized recommendations were justified. Discussion centered around which recommendations to address first including the extension of maternity leave for the formal sector, providing financial support for maternity leave for the informal sector, building capacity by investing in training lactation specialists in Samoa, breastfeeding education and promotion in the community, and strengthening workplace environment to become breastfeeding friendly.

Decision makers demonstrated their commitment to strengthen breastfeeding in Samoa in two ways. First, the director general signed a revised and updated breastfeeding policy for hospitals and it was launched at this event. Second, various ministries including MOH, MCIL, MESC, agreed to begin with implementing the most feasible recommendations while also planning for those that would require a longer implementation timeframe. Decision makers thus committed to implementing one resolution that was deemed as most feasible. Decision makers renewed their commitment to implement a 2011 mandate instructing each ministry to establish a lactation room that provided a refrigerator for breastmilk, pumping station, and chair to allow mothers a safe environment to breastfeed. A representative from the MCIL put forth the suggestion to create an environment for their workplace resulting in an agreement across ministries to create breastfeeding friendly workplaces through the implementation and enforcement of breastfeeding spaces within government ministries. The MCIL and MOH elected to pilot the program and provide funding from their budget with the goal of being the role model for other ministries to move forward with establishing lactation rooms for their employees. It was decided that once all ministries had lactation rooms, the private sector would be approached to being working with them to establish breastfeeding friendly environments within their workspaces. To date, the MOH has established a lactation room within the Ministry and is working to establish others.

## Discussion

For Samoa, implementing BBF has demonstrated their further commitment to strengthening their breastfeeding friendly environment. For the first time, BBF brought together a diverse group of stakeholders from across Samoa invested in strengthening the breastfeeding environment in the country. The gathering of stakeholders to dialogue and reach consensus on the top recommendations is an essential process to garner support, cooperation, and lead to the acceptance and implementation of the recommendations. Within Samoa, this process led to the full support of six priority recommendations to strengthen the breastfeeding friendly environment, increasing the likelihood that there would be movement towards policy implementation. Indeed, it was at the launching of the BBF recommendations that the updated breastfeeding policy was signed after years of trying to garner support from all key parties. To date, the signing of the breastfeeding policy along with BBF committee network efforts has led to the achievement of 80% of the BFHI Ten Steps in the two main hospitals in Samoa. These outcomes document the value of BBF across Samoa and is echoed among all other pretested BBF countries.

The implementation of BBF in Samoa identified clear key gaps within the breastfeeding environment that required strengthening to initiate the scaling up of breastfeeding policy and programs in the country. Samoa’s lack of a specific budget line assigned to breastfeeding programs is not unique as other countries, including Mexico [11] and Ghana [12], have demonstrated similar results. Funding and resources are needed to drive the training of health care providers to deliver high-level breastfeeding support and education plus promote breastfeeding through various channels [8]. However, this financial commitment to national breastfeeding programs must come from the government to help countries try to reduce reliance on external funding to sustain breastfeeding programs [8].

Samoa emerged with a rich set of priority recommendations aimed at strengthening their breastfeeding friendly environment. Similar to Mexico [11], the resolution that decision makers felt was most feasible to first commit to was the establishment of lactation rooms within the public sector, primarily ministries. Improving the workplace environment has been shown to improve exclusive breastfeeding rates, with more services increasing rates in a dose-response manner [22]. By taking a first step to strengthen the workplace environment, Samoa is demonstrating their commitment to strengthening maternity protection; an action which has the potential to encourage additional changes that can reap long-term rewards in promoting breastfeeding and improving breastfeeding outcomes.

Several lessons were learned during the implementation of BBF in Samoa. First, for countries planning to implement BBF, an in-country director is needed to lead and coordinate BBF [9]. Therefore, it is essential to include BBF into the director’s annual workplan so that is a planned responsibility rather than an added one. Within Samoa, BBF was an added responsibility thus the director found it challenging to set aside time to fully implement BBF given the additional duties and roles that person was responsible for within the MOH. While support staff provided technical assistance to organize meetings, develop policy briefs and infographics, and collect data, the time commitment required by the director to lead BBF was extensive.

Second, building on Ghana’s experience [12], having motivated breastfeeding experts who can devote the time to implementing BBF was essential to completing BBF within Samoa. Within Samoa, BBF committee members were highly diverse in their backgrounds as well as expert in one of the key gears, subsequently they were able to provide strong support, such as guidance and data, for the gears. Additionally, members were supportive of breastfeeding initiatives and thus motivated to complete BBF accurately and on-time. This supports the BBF guidance that selecting committee members is an essential component leading to the successful implementation of BBF [9].

Third, more gear team meetings may be needed to assist with the transfer of information and data plus to ensure accurate scoring of benchmarks and gears. Within Samoa, the operational manual was expansive and challenging for committee members to familiarize themselves with and use. Additionally, committee members were limited in their ability to access the BBF Dropbox (used to share data and scoring between gear members and the director) due to weak or no internet access at their workplaces. Frequent gear team meetings helped assure that the benchmarks were understood, the needed data was collected and shared, and scoring was accurate.

Fourth, continual information sharing ensured that high level decision makers were informed throughout the BBF process. The director shared information with gear teams through email groups, which ensured all gear members and their respective organizations plus the heads of governments ministries were informed about the progress of the BBF committee meetings and resolutions throughout the process. This too supports the BBF guidance that continual engagement of decision makers throughout the process is critical to the implementation of BBF recommendations [9].

## Conclusion

In conclusion, the implementation of BBF in Samoa yielded some similar results as Mexico and Ghana. Decision makers identified similar areas to initially target and strengthen the breastfeeding environment plus some common lessons were learned. Samoa did show there was a need to adapt BBF to meet specific needs including time and technology challenges, yet these are not context specific and can be considered within countries facing these limitations. Such results have important implications as Samoa’s successful implementation of BBF indicates that this initiative can be applied to other countries within the Western Pacific region that are also motivated to strengthen their breastfeeding environment.

## Data Availability

The datasets analyzed during the current study are available from the corresponding author on reasonable request.

## Abbreviations

BBF: Becoming Breastfeeding Friendly
BFGM: Breastfeeding Gear Model
BFHI: Baby Friendly Hospital Initiative
MCIL: Ministry of Commerce, Industry, and Labor
MESC: Ministry of Education, Sports, and Culture
MOH: Ministry of Health
MWCSD: Ministry of Women, Community, and Social Development
PSC: Public Service Commission
SQA: Samoa Qualification Authority
